# Electroacupuncture Pretreatment Ameliorates Perioperative Neurocognitive Disorder in Aged Mice by Inhibiting Ferroptosis Through the SIRT1/NRF2/GPX4 Pathway

**DOI:** 10.1111/jcmm.71021

**Published:** 2026-01-13

**Authors:** Zhongying Du, Binsen Zhang, Tianren Chen, Chang Lei, Lu Tang, Sasa Yang, Chunai Wang

**Affiliations:** ^1^ The First Clinical Medical College Gansu University of Chinese Medicine Lanzhou China; ^2^ Key Laboratory of Gansu Provincial Prescription Mining and Innovative Translational Laboratory Lanzhou China; ^3^ Gansu Provincial Traditional Chinese Medicine New Product Creation Engineering Laboratory Lanzhou China; ^4^ Gansu Provincial Hospital of Traditional Chinese Medicine Lanzhou China; ^5^ Clinical Medical Research Centre for Integrated Chinese and Western Medicine in Anesthesia of Gansu Provincial Lanzhou China

**Keywords:** electroacupuncture, ferroptosis, perioperative neurocognitive disorder, SIRT1/NRF2/GPX4

## Abstract

Perioperative neurocognitive disorder (PND) is a common complication after anesthesia surgery in elderly patients, which not only reduces the patients' quality of life but also increases the burden on their families and society. PND has been found to be closely related to ferroptosis. This study investigated whether electroacupuncture (EA) can inhibit ferroptosis through the SIRT1/NRF2/GPX4 pathway to improve PND in aged mice. The PND model was established using sevoflurane anesthesia and tibial fracture surgery. EA was administered at the Baihui (GV 20) and Dazhui (GV 14) acupoints. Additionally, intraperitoneal injection of silent information regulator sirtuin 1 (SIRT1) inhibitor EX527 (5 mg/kg) was administered for five consecutive days before surgery and intraperitoneal injection of ferrostatin‐1 (Fer‐1) (2 mg/kg) was administered before anesthesia. On the third day after surgery, the cognitive ability of the aged mice was measured using the Y‐maze, and motor ability was assessed by total distance in the open field test. Transmission electron microscopy was used to observe hippocampal mitochondrial structure. Immunofluorescence staining was used to detect glutathione peroxidase 4 (GPX4) levels in the hippocampus. Flow cytometry measured ATP content and mitochondrial membrane potential in hippocampal mitochondria. A colorimetric assay was used to detect iron content in hippocampal neurons. Reverse transcription‐quantitative polymerase chain reaction and Western blotting were used to detect mRNA and protein expression of Solute carrier family 7 member (SLC7A11), transferrin receptor 1 (TFR1), iron regulatory protein 2 (IRP2), ferritin, SIRT1, nuclear factor erythroid 2‐related factor 2 (NRF2) and GPX4. The results showed that compared with the model group, the EA treatment group and the Fer‐1 (iron inhibitor) treatment group revealed improved ferroptosis and memory function in hippocampal neurons, while the EX527 (SIRT1 inhibitor) treatment group did not reveal any improvement. In conclusion, the occurrence and progression of PND are closely related to ferroptosis. EA stimulation of the Baihui and Dazhui acupoints can improve PND, possibly by regulating ferroptosis through the SIRT1/NRF2/GPX4 signalling pathway.

## 1 Introduction

Perioperative neurocognitive disorder (PND) is one of the common postoperative complications in elderly patients, mainly including acute postoperative delirium, delayed neurological recovery and postoperative cognitive dysfunction [1, 2, 3]. It is clinically characterised by memory and cognitive impairment, as well as personality changes [4]. According to the study, the probability of PND in older patients undergoing noncardiac surgery is 30% in the first week after surgery, 10% in the first month after surgery and 1% in the first year after surgery [5]. At present, the pathogenic factors of PND remain unclear, and advanced age is the only established risk factor [6, 7, 8]. PND is closely related to postoperative neurodegenerative diseases in elderly patients [7, 9, 10], which significantly reduce the patients' quality of life and increase the care costs and the economic burden on patients' families and society [11, 12, 13]. Therefore, exploring a prevention and treatment strategy that is both efficient and cost‐effective is of great clinical significance and social value in improving the postoperative health of elderly patients and reducing the societal healthcare burden.

Ferroptosis is a novel mode of cell death, mainly caused by an imbalance between cellular oxidative stress and the antioxidant system, disturbed iron metabolism and accumulation of iron‐dependent lipid peroxides [14, 15]. As an important site of anti‐oxidative stress, mitochondria play an important role in ferroptosis and ferroptosis is associated with cell dysfunction [16, 17, 18]. Studies have demonstrated that after sevoflurane anaesthesia and surgery, mitochondrial morphology and structure change, function is reduced, antioxidant capacity is decreased and ferroptosis occurs in hippocampal neuronal cells, leading to cognitive decline in mice [19].

The silent information regulator sirtuin 1 (SIRT1) protein is a highly conserved NAD+‐dependent deacetylase that belongs to the sirtuin family [20, 21]. The deacetylation mediated by SIRT1 has an important impact on cell aging, apoptosis, glucose and lipid metabolism, oxidative stress and inflammation [22, 23, 24]. SIRT1 downstream factors include nuclear factor erythroid 2‐related factor 2 (NRF2) and glutathione peroxidase 4 (GPX4), which are important transcription factors that regulate the intracellular antioxidant stress response and are closely associated with neurological disorders, autoimmune diseases and other diseases [25, 26].

Electroacupuncture (EA), as a product of the combination of traditional and modern medicine, is characterised by low cost, significant effect, simplicity and convenience, and it plays a role in preventing and treating various diseases in daily life. Studies have indicated that EA provides neurotransmitter protection, protects mitochondrial function, reduces inflammation and has antioxidant effects [27, 28]. Animal studies have demonstrated that acupuncture at the Baihui acupoint in mice protects telomerase activity, reduces oxidative damage, decreases neuroinflammation and autophagy dysfunction and improves cognitive ability after surgery in aged mice [29]. Wang et al. indicated that pretreatment with EA improved cognitive dysfunction, increased telomerase activity and TERT protein expression, and reduced oxidative damage, autophagy dysfunction and neuroinflammation in aged mice [30]. Clinical studies have indicated that EA preconditioning improves IL‐1β and IL‐6 levels in patients' postoperative serum, reduces neuroinflammation and improves cognitive impairment [31, 32]. All of the above studies indicate that EA has a certain preventive effect on postoperative cognitive impairment. Our previous results revealed that EA could protect the structure and function of mitochondria and inhibit the inflammatory and oxidative stress responses in the hippocampus through the SIRT1 energy metabolism‐related signalling pathway to alleviate cognitive impairments after anaesthesia surgery in rats [33]. Whether EA could reduce the ferroptosis of hippocampal neuronal cells through modulation of the SIRT1/NRF2/GPX4 signalling pathway, protect mitochondrial function and energy metabolism, attenuate oxidative stress and ameliorate the occurrence of PND in aged mice deserves to be explored in further studies.

This study aimed to establish a PND model by sevoflurane anaesthesia and tibial fracture surgery and to intervene in PND mice using EA, the SIRT1 inhibitor EX527 and the ferroptosis inhibitor Fer‐1 to elucidate whether EA could modulate ferroptosis through the SIRT1/NRF2/GPX4 signalling pathway and thus improve perioperative cognitive dysfunction in aged mice.

## 2 Materials and Methods

### 2.1 Animals

Sixty healthy male C57/BL6 mice (15 months old, weighing 32–35 g) purchased from SPF (Beijing) Biotechnology Co. Ltd. were housed in a standard SPF‐grade laboratory (temperature 23°C ± 2°C, 12‐h light/dark cycle, humidity 50% ± 10%), with access to a standard diet and water. After 1 week of acclimatisation, the mice were divided into six groups using a random number table method: control group (C, *n* = 10), model group (M, *n* = 10), model + EA group (E, *n* = 10), model + Ferrostatin‐1 (Fer‐1) group (F, *n* = 10), model + EA + Ferrostatin‐1 (Fer‐1) group (EF, *n* = 10) and model + EA + EX527 group (EX, *n* = 10), with EX527 being a SIRT1 inhibitor. The procedures and protocols used in this study were approved by the Animal Ethics Committee of Gansu University of Traditional Chinese Medicine, Ethics No. 2023‐512.

### 2.2 Surgery and Anaesthesia

The mice were fasted for 8 h before anaesthesia and placed in the same anaesthesia induction box during the procedure. They were administered 3% sevoflurane by inhalation with a 50% air‐oxygen mixture as a carrier. Aseptic open intramedullary fixation of a tibial fracture was performed after induction of anaesthesia. The right hind paw was selected, shaved and sterilised. The skin was incised below the knee joint to expose the tibia, and an intramedullary fixation pin was drilled and implanted through the tibial transposition. The incision was then sutured, and lidocaine (2%) was topically applied to manage postoperative pain. The concentration of anaesthesia gas and oxygen in the anaesthesia box was monitored by a gas monitor, and sevoflurane anaesthesia was maintained for 2 h. After anaesthesia was terminated, pure oxygen was inhaled for 15 min before the mice were returned to their cages after natural awakening. In the control group, the mice were placed in a transparent anaesthesia induction box with a 50% air‐oxygen gas mixture for 2 h, received an intraperitoneal injection of an equal amount of saline and were then returned to their cages for free feeding.

### 2.3 EA and Medication

Groups E, EF and EX were treated with EA at the Baihui acupoint (GV 20, the intersection of the midline of the parietal bone and the anterior borders of both ears in mice) and the Dazhui vertebra acupoint (GV 14, between the spinous process of the seventh cervical vertebra and the intervertebral disc of the first thoracic vertebra), selected according to a previous study and the acupoint location was referred to in the ‘Names and Location of Common Acupoints for Experimental Animals Part 3: Mice T/CAAM 0002‐2020’ issued by the Chinese Acupuncture and Moxibustion Society in 2020 [34]. Four days before the operation and on the day of the operation, EA was performed at the same time using a 30‐gauge Millipore needle (5 mm long, 0.18 mm in diameter, Shenzhen Kester Metal Products Co. Ltd., Shenzhen, China). The needle at the Baihui acupoint was inserted at a 45° angle to the epidermis, approximately 2 mm subcutaneously. At the Dazhui acupoint, the needle was inserted vertically to a depth of about 5 mm. EA stimulation was applied using the SDZ‐V EA instrument (Shanghai Huatuo, China) with sparse‐dense waveforms, frequency of 2/15 Hz and intensity of 1 mA for 30 min once per day. Ferrostatin‐1 (Fer‐1) at a dose of 2 mg/kg was administered intraperitoneally to groups F and EF before anaesthesia. EX527, a SIRT1 inhibitor, was administered intraperitoneally at a dose of 5 mg/kg to group EX mice on the 4 days prior to surgery and again on the day of anaesthesia for the operation.

### 2.4 Open Field Test (OFT)

Mice were subjected to an OFT on the third day after anaesthesia surgery. Mice were placed in a square area measuring 40 × 40 × 40 cm and allowed to move freely for 5 min. The trajectories of the mice were tracked and recorded, and the total distance travelled was used to assess locomotor ability. After each test, the device was wiped with 75% ethanol to eliminate olfactory cues. The testing environment was kept quiet to avoid disturbing the mice.

### 2.5 Y‐Maze Test

The Y‐maze system was used to assess the memory ability of the mice. The three arms of the Y‐maze were randomly divided into the novel arm, the start arm and the other arm. The experiment consisted of two main phases: the training phase and the testing phase. During the training phase, the novel arm was blocked by a partition, and the mice were placed in the start arm and allowed to explore the start and the other arms freely for 10 min. After training, the mice were returned to their home cages for 1–2 h. In the detection phase, the partition blocking the novel arm was removed. The mice were again placed in the start arm and allowed to explore all three arms freely for 5 min. Video recordings were used to track the time each mouse spent in each arm, as well as the number of entries and distance travelled by each mouse in each arm during the 5‐min period. Mice with intact spatial reference memory would explore the novel arm more frequently. The Y‐maze was thoroughly cleaned with 70% alcohol after each mouse in both phases to minimise the influence of residual odours on subsequent mice.

### 2.6 Transmission Electron Microscope (TEM)

After removing the brain, the specimen was fixed with tissue fixative. The hippocampal tissue was cut into squares smaller than 1 mm^3^ and fixed in 2.5% glutaraldehyde for 2–4 h. After dehydration, infiltration, embedding and curing, the samples were sliced into sections 60–80 nm thick. The ultrastructure of the hippocampus was then observed under an electron microscope after double‐staining with 2% uranyl acetate lead citrate.

### 2.7 Immunofluorescence Staining

Mouse brains were collected by cardiac perfusion with saline followed by 4% paraformaldehyde at the end of the behavioural studies. Specimens were fixed in 4% paraformaldehyde for 24–48 h, then dehydrated in ethanol and embedded in paraffin. Coronal sections (3.5 μm thick) were prepared. After deparaffinisation and antigen retrieval, the slides were drained for a few seconds, and the area around the tissue was dried using filter paper. The tissue was outlined with an immunohistochemistry pen (ensuring the circle was closed at both ends and not drawn onto the tissue). Drops of primary antibody NeuN (1:200) and primary antibody GPX4 (1:200) were added until the tissue was completely covered. Multicolor immunofluorescence staining was performed using the Triple Fluorescence Immunohistochemistry Mouse/Rabbit Kit (RS0036; Immunoway) according to the manufacturer's protocol. Immunofluorescence images were captured using a McAudi scanner (Mikroscan Fujian, China), and images of the hippocampal region were acquired and analysed for the number of GPX4‐positive cells co‐labelled with NeuN.

### 2.8 Mitochondrial Membrane Potential (MMP) Levels and ATP Content

The MMP assay kit JC‐1 (Beijing Solarbio Science & Technology Co. Ltd.) and the ATP content assay kit (Beijing Solarbio Science & Technology Co. Ltd.) were used following the manufacturer's instructions. MMP and ATP levels in hippocampal neurons were measured using flow cytometry (BD, LSRFortessa) and an enzyme reader (Thermo Scientific, Multiskan FC) according to the manufacturer's instructions.

### 2.9 Iron Content

For each group, 50 mg of mouse hippocampal tissue was collected, and 0.5 mL of extraction solution was added. Samples were homogenised in an ice bath. The homogenates were centrifuged at 4°C, 12,000 rpm for 5 min, and the supernatant was collected. The Fe^2+^ concentration in hippocampal tissue was determined using the Iron Content Test Kit (Ferrozine Colorimetric Assay), following the manufacturer's instructions.

### 2.10 Reverse Transcription‐Quantitative Polymerase Chain Reaction (RT‐qPCR)

Hippocampal tissues were collected from each group, and total RNA was extracted using the RNA Extraction Kit (Yeasen MolPure cell/Tissue Total RNA Kit, Cat: 19225650) according to the instructions. First‐strand cDNA was synthesised using the Hifair III1st Strand cDNA Synthesis SuperMix for qPCR (Yeasen Biotechnology, Cat: 1114ES60) according to the instructions. The qPCR was then performed using Hieff UNICON Universal Blue SYBR Green Master Mix (Yeasen Biotechnology, Cat: 11184ES08). A 10 μL reaction system was established on the LightCycler 96 system for real‐time PCR. The reaction conditions were: pre‐denaturation at 95°C for 5 min, denaturation at 95°C for 10 s, annealing/extension at 60°C for 30 s, repeated for 45 cycles. The expression levels of target genes were normalised to GAPDH and calculated using the 2^−ΔΔCt^ method. Primers were synthesised by Bioengineering (Shanghai) Co. The primer sequences are shown in the table below (Table 1).

**TABLE 1:** Sequences of mice‐specific primers used in RT‐qPCR for SIRT1, NRF2, GPX4, SLC7A11, TFR1, IRP2, Ferritin and GAPDH.

Genes	Primers
SIRT1	F:5′‐TCGGCTACCGAGGTCCATA‐3′
	R:5′‐CGCCTTTGGTGGTTCTGAAAGG‐3′
Nrf2	F:5′‐AAGCACAGCCAGCACATTCTCC‐3′
	R:5′‐TGACCAAGGACTCACGGGAACTTC‐3′
GPX4	F:5′‐CCCGATATGCTGATGGTGGTTTAC‐3′
	R:5′‐ATTTCTTGATTACTTCCTGGCTCCTG‐3′
SLC7A11	F:5′‐CCACCATCAGTGCGGAGGAG‐3′
	R:5′‐GAAGCAGGAGAGGGCAACAAAG‐3′
TFR1	F:5′‐GGAGTCTCCCGAGGGTTATGT‐3′
	R:5′‐CACCAGTTCCTAGATGAGCA‐3′
IRP2	F:5′‐CTTTTTCCCTGCCCGTGTTG‐3′
	R:5′‐AACACTGTCTCAGGTCATTTACT‐3′
Ferritin	F:5′‐CAAGTGCGCCAGAACTACCA‐3′
	R:5′‐GTCATCACGGTCTGGTTTCTTTAT‐3′
GAPDH	F:5′‐GGTTGTCTCCTGCGACTTCA‐3′
	R:5′‐TGGTCCAGGGTTTCTTCC‐3′

### 2.11 Western Blotting (WB)

Total protein was extracted by placing the hippocampal tissue into a lysis mixture containing phosphatase and protease inhibitors. The protein concentration was determined using the BCA protein assay kit (Beijing Solarbio Science & Technology). According to the molecular weight of the target proteins, different concentrations of separator gel were prepared. A 20 μg sample of protein was loaded into each well, electrophoresis was performed at 80 V and after the marker separated, the voltage was increased to 120 V. After electrophoresis, proteins were transferred to a PVDF membrane. The membrane was blocked with a rapid blocking solution (New Cell & Molecular Biotech) for 10 min at room temperature and washed three times with TBST for 10 min each. The membrane was then incubated overnight at 4°C with primary antibodies: SIRT1 (1:850; Lot‐19G10A10; BOSTER), NRF2 (1:1500; Cat#YT3189; Immunoway), iron regulatory protein 2 (IRP2) (1:3000; Cat#YN3307; Immunoway), transferrin receptor 1 (TFR1) (1:750; LotNo‐23BP65E1; BOSTER), GPX4 (1:1500; Cat#YN3047; Immunoway), ferritin (1:3000; Cat#YT1692; Immunoway) and SLC7A11 (1:2000; Cat#YT8130; Immunoway). After incubation, the membrane was washed three times with TBST for 10 min each, then incubated with an HRP‐conjugated secondary antibody (anti‐mouse or anti‐rabbit IgG) (1:10,000; Cat#RS0001, Cat#RS0002; Immunoway) for 1 h at room temperature. ECL luminescent solution was added, and the membrane was exposed for imaging. After imaging, the membrane was regenerated using a membrane regeneration solution and incubated with the GAPDH antibody (1:5000, Cat#YM3029, Immunoway) following the same steps as before. Protein bands were visualised using a gel imaging system (GeLView 6000Plus) and analysed using Image J analysis software.

### 2.12 Statistical Analysis

GraphPad Prism Software (version 9.5.1) was used for statistical analysis and graphing. Quantitative data is expressed as mean ± standard error of the mean (SEM). One‐way analysis of variance (ANOVA) was used for analysis, with comparisons between two groups made using Tukey–Kramer's multiple comparison test or Student's unpaired *t*‐test. Data were tested for normality using the Shapiro–Wilk test before statistical analysis. Comparisons of pre‐ and post‐treatment data within the same groups in the Y‐maze were analysed using two‐way ANOVA followed by Bonferroni post hoc test. A *p* < 0.05 was considered statistically significant.

## 3 Results

### 3.1 Mouse Mobility Is Unaffected by Surgery

**FIGURE 1:**
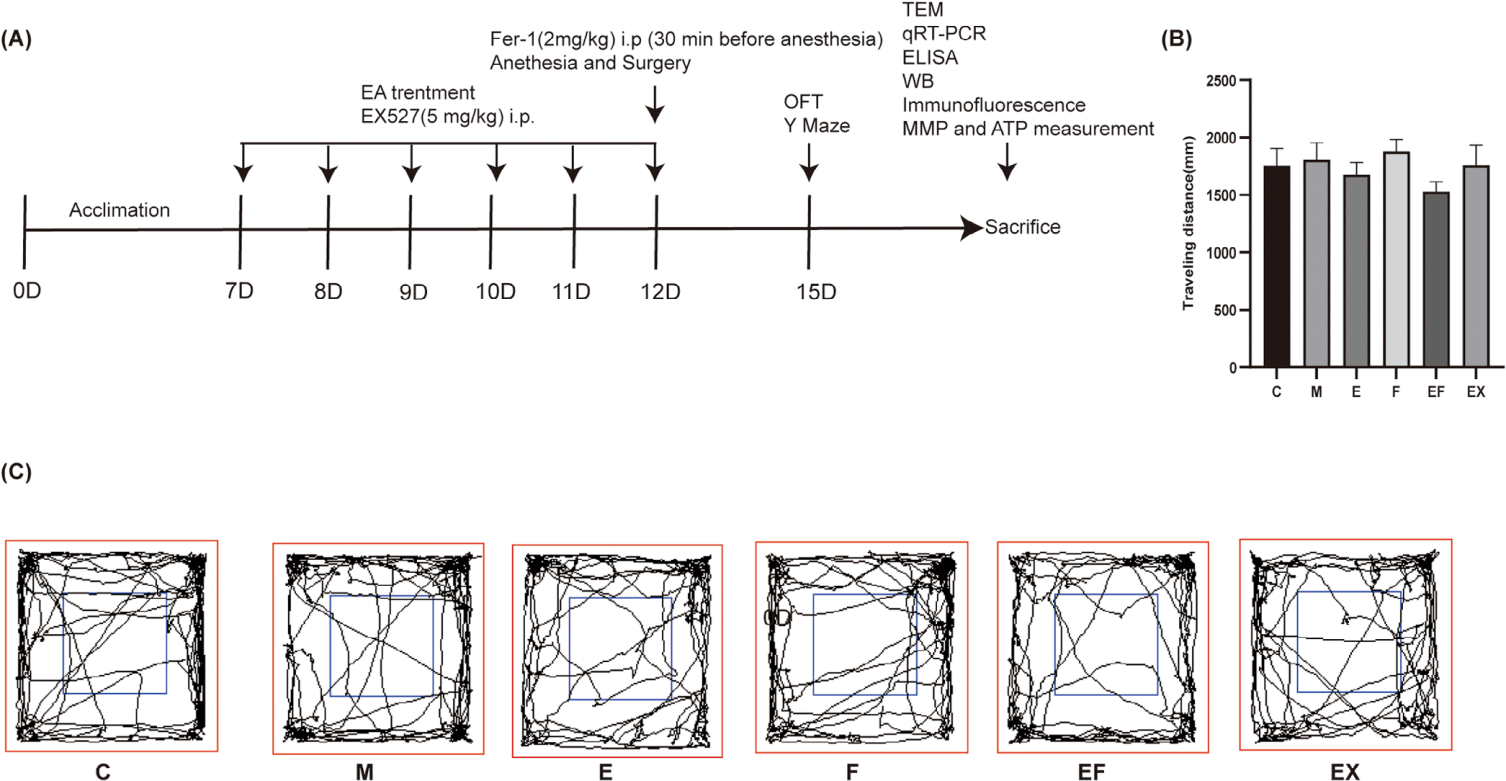
Flowchart of the experiment and the spontaneous locomotor activity remained unaffected after surgery in aged mice. (A) Flowchart of the experiment. (B) Distance of travelling. (C) Travelling trajectory in the OFT (*n* = 6 per group).

To exclude the effect of internal fixation surgery for tibial fracture on the locomotor activity of mice, we performed an OFT experiment where the total walking distance was used to assess locomotor activity. The results revealed no significant difference in total distance between each group (Figure 1, *p* > 0.05), suggesting that the impaired behavioural performance of the mice was not caused by the surgery.

### 3.2 EA Pretreatment Alleviates Cognitive Decline in PND Mice Caused by Ferroptosis of Hippocampal Neurons

**FIGURE 2:**
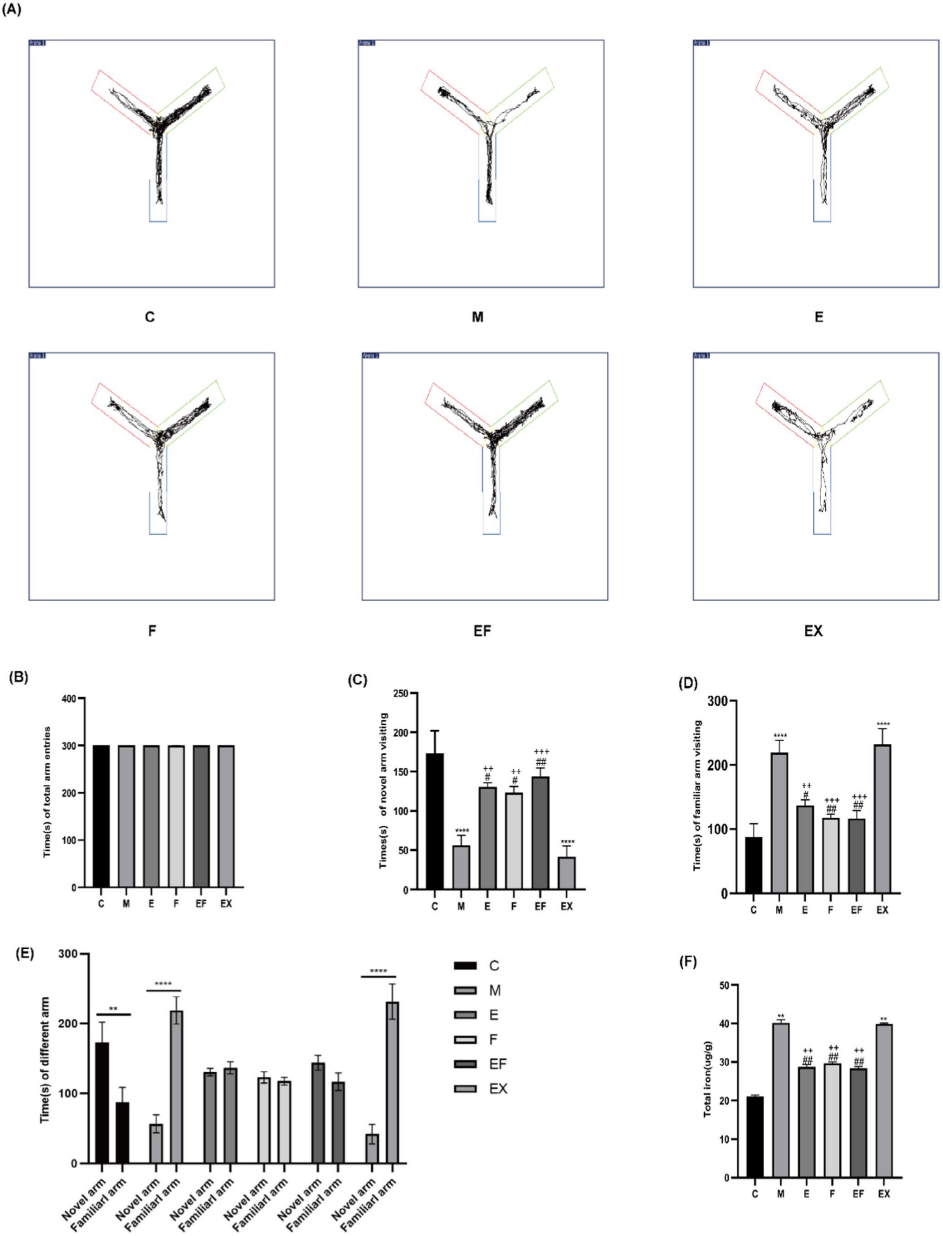
Neuronal ferroptosis‐induced cognitive deficits. (A) Y‐maze trajectory map. Red arm: Start arm; Green arm: Novel arm; Blue arm: Other arm. (B) Time (s) of total arm entries (*n* = 6 per group). (C) Time (s) of novel arm visits (*n* = 6 per group). (D) Time (s) of familiar arm visits (*n* = 6 per group). (E) Time (s) spent in different arms (*n* = 6 per group). (F) Total iron content in the hippocampus (*n* = 3 per group). Values are presented as mean ± SEM. **p < 0.01 and ****p < 0.0001 compared with the C group. #*p* < 0.05 and ##*p* < 0.01 compared with the M group; ++*p* < 0.01 and +++*p* < 0.001 compared with the EX group. Data were analysed using two‐way ANOVA followed by Bonferroni post hoc test.

To test our hypothesis that EA pretreatment could alleviate cognitive decline induced by neuronal ferroptosis in PND mice, a Y‐maze test was conducted to evaluate the learning and memory capabilities of the mice (Figure 2A). The results of hippocampal iron content (Figure 2F) demonstrated that hippocampal iron content was significantly reduced in group C and group E compared with group M, suggesting that Sevoflurane anaesthesia led to ferroptosis of hippocampal nerve cells and EA pretreatment alleviates sevoflurane‐induced hippocampal iron accumulation in aged mice. The results of the Y‐maze experiment revealed no statistically significant differences in the total time spent entering the arms among the groups (Figure 2B). Compared with E, F and EF groups, the M group exhibited reduced time entering the novel arm (Figure 2C) and increased time entering the familiar arm (Figure 2D). When comparing the time spent entering the two arms within the same groups (Figure 2E), the total time spent entering the familiar arm in the M group was longer than that spent entering the novel arm. Conversely, in E, F and EF groups, the total time spent entering the familiar arm was shorter than that spent entering the novel arm. These findings suggest that EA treatment can effectively mitigate the decline in learning and memory abilities of PND mice induced by neuronal ferroptosis.

### 3.3 EAPretreatment Ameliorates Ferroptosis and Mitochondrial Dysfunction in Hippocampal Neurons Induced by Sevoflurane Anaesthesia in Aged Mice

**FIGURE 3:**
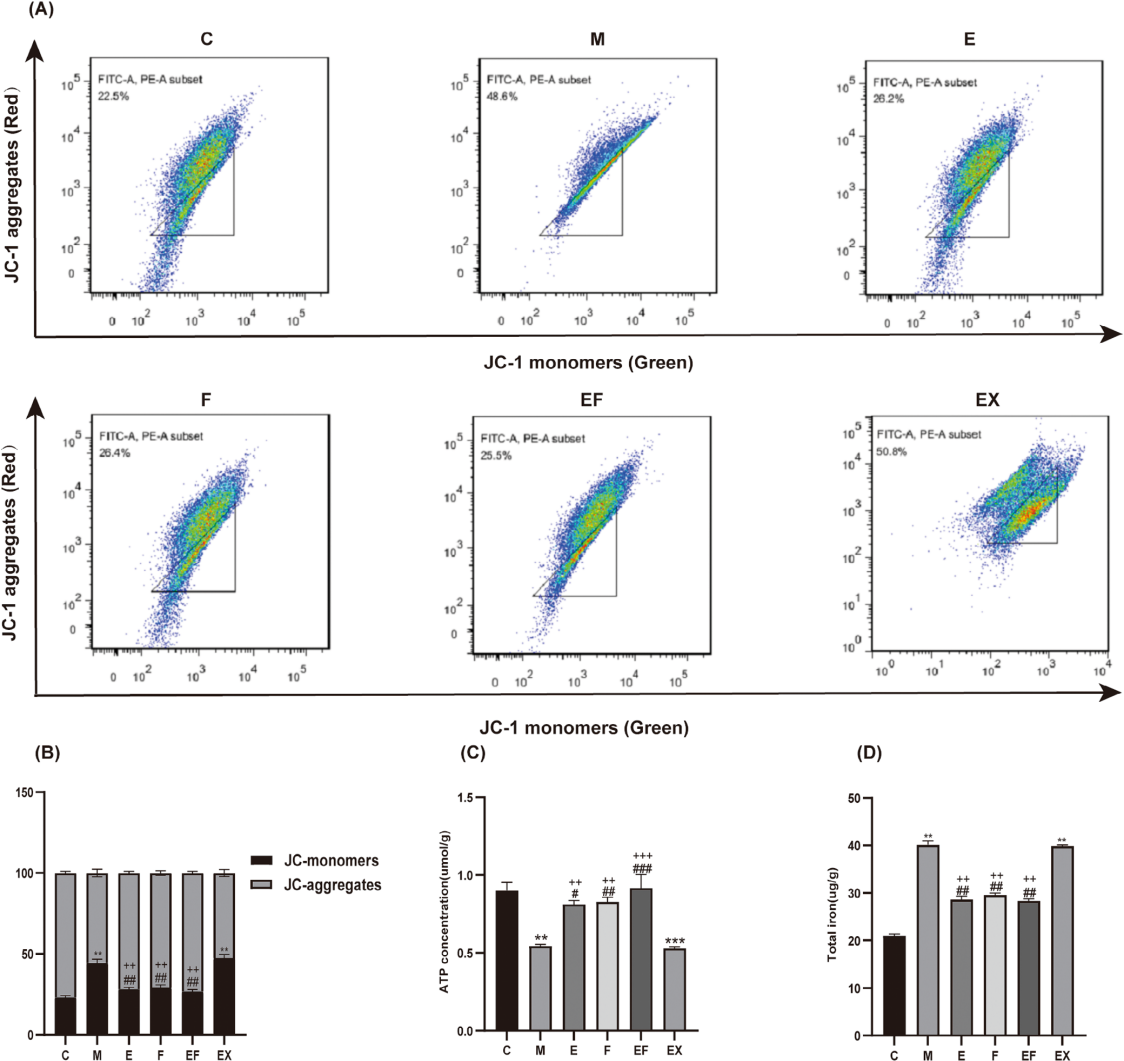
Sevoflurane anaesthesia induces ferroptosis and mitochondrial dysfunction in the hippocampus of aged mice. (A) JC‐1 staining assay (*n* = 3 per group). (B) Analysis of MMP by JC‐1 staining assay. (C) ATP content in the hippocampus. (D) Iron content in the hippocampus (*n* = 3 per group). Values are presented as mean ± SEM. **p < 0.01 and ***p < 0.001 compared with the C group; #*p* < 0.05, ##*p* < 0.01 and ###*p* < 0.001 compared with the M group; ++*p* < 0.01 and +++*p* < 0.001 compared with the EX group.

To investigate the effect of EA pretreatment on hippocampal iron content in aged mice following sevoflurane anaesthesia, the iron content in hippocampal cells was measured. The results demonstrated that hippocampal iron content was significantly reduced in groups E, F and EF compared to group M, suggesting that EA pretreatment alleviates sevoflurane‐induced hippocampal iron accumulation in aged mice (Figure 3D). Additionally, MMP levels and ATP content were assessed. The findings revealed that MMP levels and ATP content were markedly increased in groups E, F and EF compared to group M (Figure 3A–C), indicating that EA pretreatment enhances mitochondrial function following sevoflurane anaesthesia.

### 3.4 SIRT1/NRF2/GPx4 Signalling Is Involved in the Ferroptosis of Hippocampal Neuronal Cells After Sevoflurane Anaesthesia Surgery

**FIGURE 4:**
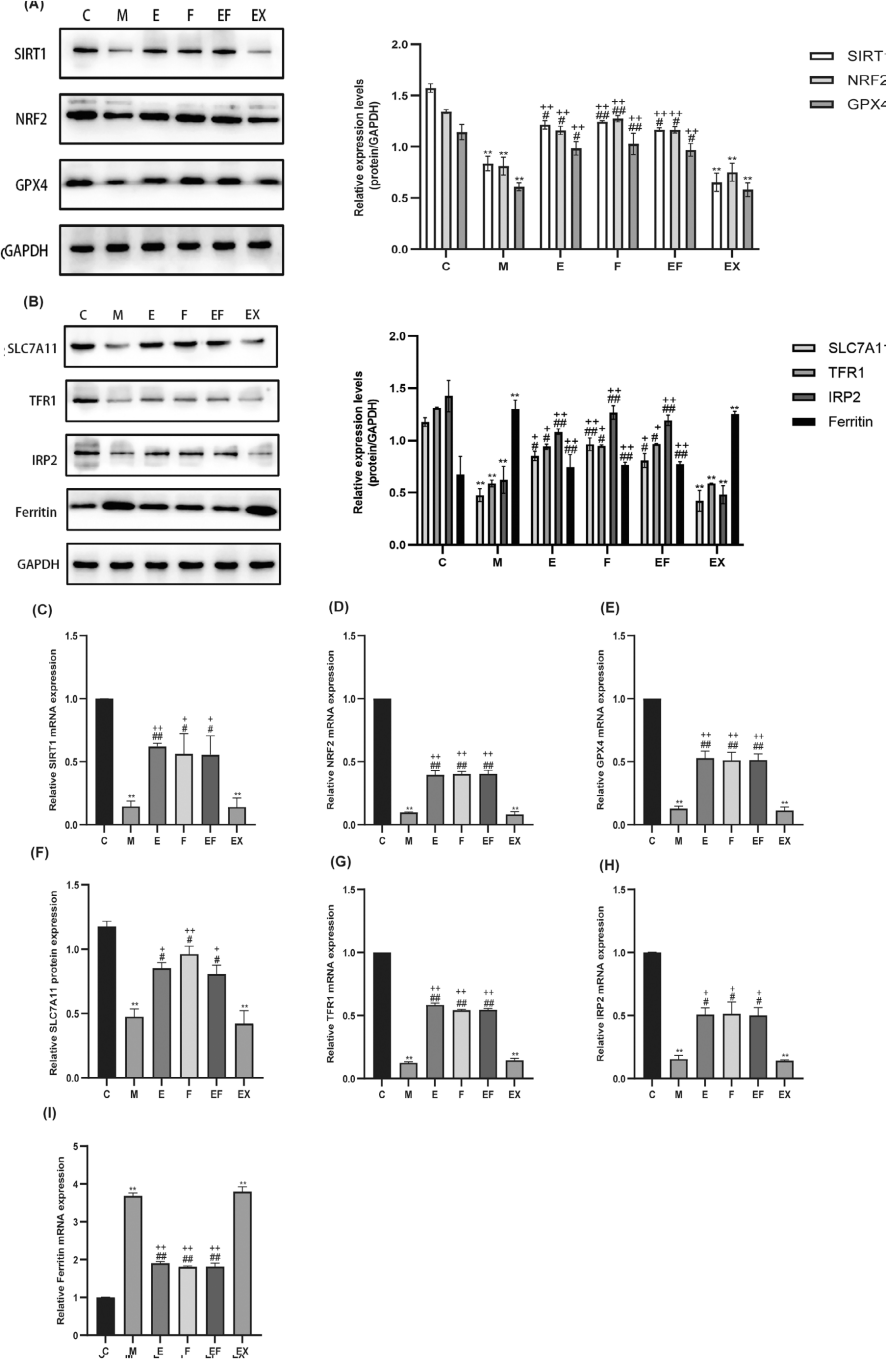
SIRT1/NRF2/GPX4 pathway is involved in hippocampal ferroptosis in aged mice. (A) WB images and quantification analysis of SIRT1, NRF2 and GPX4 in the hippocampus of aged mice. (B) WB images and quantification analysis of SLC7A11, TFR1, IRP2 and ferritin in the hippocampus of aged mice (*n* = 3 per group). (C) qRT‐PCR expression of SIRT1, NRF2, GPX4, SLC7A11, TFR1, IRP2 and ferritin mRNA in the hippocampus of aged mice (*n* = 3 per group). Values are presented as mean ± SEM. **p < 0.01 compared with the C group; #*p* < 0.05 and ##*p* < 0.01 compared with the M group; +*p* < 0.05 and ++*p* < 0.01 and compared with the EX group.

Cells maintain intracellular iron homeostasis through the action of iron regulatory proteins (IRP1 and IRP2). Upon an increase in intracellular iron levels, cells are prone to undergo ferroptosis. TFR1 mediates iron uptake, while ferritin serves as the primary protein for iron storage. To explore the role of the SIRT1/NRF2/GPX4 signalling pathway in hippocampal ferroptosis following sevoflurane anaesthesia, we employed the SIRT1 inhibitor EX527 and the iron chelator Fer‐1 as interventions. The protein expression levels of SIRT1, NRF2, GPX4, IRP2, TFR1 and ferritin in the mouse hippocampus, as well as their corresponding mRNA levels, were assessed using WB and RT‐PCR analyses. The results demonstrated that compared with the control group (C group), the protein expressions of SIRT1, NRF2, GPX4, IRP2 and TFR1 were significantly reduced in the M, EX and EF groups (Figure 4A–E,G,H), whereas the expression of ferritin was markedly increased (Figure 4B,I) following treatment with EX527 and Fer‐1 inhibitors. These findings suggest that the SIRT1/NRF2/GPX4 signalling pathway plays a critical role in sevoflurane‐induced hippocampal ferroptosis.

### 3.5 EAPretreatment Ameliorates the Ferroptosis of Hippocampal Neuronal Cells Induced by Sevoflurane Anaesthesia Through the SIRT1/NRF2/GPX4 Signalling Pathway

**FIGURE 5:**
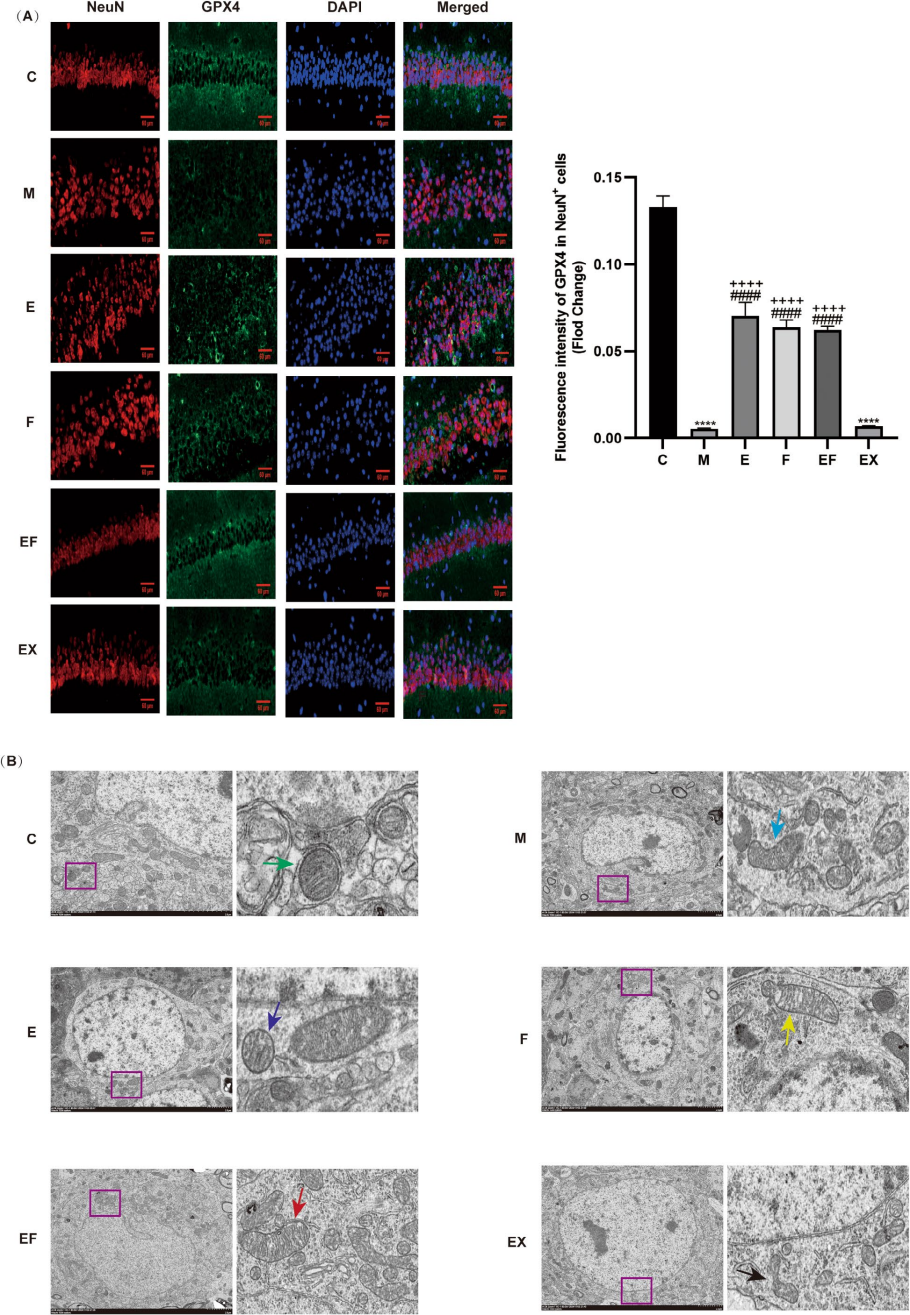
EA ameliorates sevoflurane aesthesia‐induced ferroptosis in the hippocampus of aged mice through the SIRT1/NRF2/GPX4 signalling pathway. (A) Representative immunofluorescence staining of GPx4 (green) and NeuN (red) in the hippocampal CA1 region and fluorescence intensity of GPX4 levels in NeuN‐positive cells quantified in each group. Scale bar = 60 μm. (B) Representative TEM images of hippocampal neuron mitochondria in different groups. Scale bar = 2 μm. Values are presented as mean ± SEM (*n* = 3 per group). ****p < 0.0001 compared with the C group; ####*p* < 0.0001 compared with the M group; ++++*p* < 0.0001 compared with the EX group.

GPX4 is extensively utilised as a marker for ferroptosis. In this study, we conducted TEM of hippocampal mitochondria and immunofluorescence staining of GPX4 in the hippocampal CA1 region. The immunofluorescence results revealed that GPX4 expression was significantly downregulated in the hippocampal CA1 neurons of M and EX groups compared to the C group. Conversely, GPX4 expression in the hippocampal CA1 neurons of F and EF groups was upregulated relative to M and EX groups (Figure 5A). TEM analysis demonstrated mitochondrial atrophy, increased membrane density, reduced cristae formation and outer membrane rupture in M and EX groups. However, mitochondrial morphology was markedly improved with EA and ferrostatin treatment (Figure 5B). Additionally, qRT‐PCR and WB analyses indicated that compared to M and EX groups, the mRNA and protein levels of SIRT1/NRF2/GPX4 and SLC7A11 were elevated in E, F and EF groups, while the mRNA and protein levels of iron metabolism‐related markers IRP2, TFR1 and ferritin were decreased (Figure 4A–I). Collectively, these findings suggest that EA can alleviate sevoflurane anaesthesia‐induced ferroptosis in hippocampal neurons through the SIRT1/NRF2/GPX4 signalling pathway, thereby protecting mitochondrial integrity.

### 3.6 EAPretreatment Ameliorates the Memory Deficits Induced by Ferroptosis of Neuronal Cells in PND Mice Through the SIRT1/NRF2/GPX4Signalling Pathway

In the results of the Y‐maze experiment, in the exploration phase, group E spent more time exploring the new arm, less time in the familiarisation arm and depicted greater memory learning compared to group M and group EX (Figure 2C,D). A comparison of the results of total time in both arms between the same groups in both phases indicated no statistical difference in the time spent entering the two arms in Group E. Group M and group EX spent more total time entering the familiarisation arm than the new arm (Figure 2E). The above results suggest that EA can ameliorate memory deficits induced by ferroptosis in neuronal cells of PND mice through the SIRT1/NRF2/GPX4 signalling pathway.

## 4 Discussion

This study suggests that perioperative cognitive dysfunction after sevoflurane anaesthesia may be due to ferroptosis of neuronal cells in aged mice. EA at the Baihui and Dazhui acupoints can protect mitochondrial structure and function and reduce ferroptosis of nerve cells, and its mechanism may be mediated through the SIRT1/NRF2/GPX4 pathway.

PND have been renamed from postoperative cognitive dysfunction and mainly include acute postoperative delirium and delayed neurocognitive recovery. Currently, the pathogenesis of PND is primarily associated with neuroinflammation, oxidative stress and autophagy dysfunction. Recent studies have indicated that the occurrence of cognitive dysfunction is related to ferroptosis of hippocampal nerve cells, which is mainly caused by an imbalance in antioxidant capacity and the accumulation of lipid peroxides [35, 36, 37]. The study by Jiang et al. suggests that 15LO2‐mediated ferroptosis may be involved in offspring neurotoxicity induced by maternal sevoflurane anaesthesia in the second trimester [38]. Wang et al. demonstrated that MEF2C expression is reduced, and lipid peroxidation and iron accumulation are increased after anaesthesia surgery, leading to ferroptosis of nerve cells and postoperative cognitive dysfunction [35]. Zhao et al. described that MIB2 depletion can aggravate ferroptosis induced by sevoflurane anaesthesia, thereby affecting cognitive function in mice [39].

In this study, we used the method of Zhou et al. to establish the PND model, which indicated that cognitive ability decreased significantly on the third day after anaesthesia surgery [5]. Reducing wound pain levels could minimise the interference of pain with the results of behavioural experiments, so we measured the cognitive ability of the mice on the third day after surgery. Baihui (GV 20) and Dazhui (GV 14) are located at the intersection of the line between the two ears and the centre of the top of the head and at the posterior neck in the depression of the spinous process of the seventh cervical vertebra, respectively. These points belong to the governor vessel, which enters the brain and is believed to regulate brain function and consciousness. Studies have indicated that EA stimulation of Baihui and Dazhui acupoints can promote oligodendrocyte regeneration and improve memory impairment in a mouse model of long‐term cerebral hypoperfusion [40]. Clinical studies have also demonstrated that EA combined with moxibustion at Baihui (GV 20) and Dazhui (GV 14) can regulate serum levels of vascular endothelial growth factor and acetylcholinesterase in patients with vascular dementia [41]. Consequently, Baihui and Dazhui were selected as the acupoints in this study.

GPX4 is a representative indicator of ferroptosis [42]. Li et al. and Wen et al. observed that GPX4 expression in the CA1 region increased after the use of iron inhibitors [43, 44]. Our immunofluorescence experiment results revealed that there was no significant difference between the effects of EA and iron inhibitors, while the expression of GPX4 decreased after the use of SIRT1 inhibitors. This further suggests that EA could ameliorate ferroptosis through the SIRT1/NRF2/GPX4 pathway. Structural changes in mitochondria are the main morphological features of ferroptosis [45]. The results of TEM revealed that mitochondrial morphology improved after EA and iron inhibitor treatment, while there was no difference between the SIRT1 inhibitor group and the model group. All these results suggest that EA can improve PND by alleviating ferroptosis of hippocampal neurons through the SIRT1/NRF2/GPX4 pathway.

Ferroptosis is a newly recognised type of programmed cell death. IRP2 and TFR1 are important molecules involved in cellular iron metabolism [46, 47]. They play a key role in maintaining intracellular and extracellular iron levels. The results of the WB experiment in this study demonstrated that the expression of IRP2 and TFR1 in the model group decreased after sevoflurane anaesthesia surgery, which was consistent with the findings of Wu et al. [19], but different from the results of Wang et al. [48], In their experiment, TFR1 was increased in ferroptosis cells, while in our study, the expression of TFR1 increased. Whether this difference is related to the fact that the cells themselves are undergoing ferroptosis and TFR1 depletion warrants further exploration. Our study has some limitations. First, this study used EA, a SIRT1 inhibitor and an iron inhibitor to investigate the relationship between EA, the SIRT1/NRF2/GPX4 pathway and ferroptosis. However, there was no significant difference in the effect of EA alone, Fer‐1 treatment alone or their combination. These results may be due to the limited sample size. In addition, while our results suggest that EA can improve PND by regulating ferroptosis through the SIRT1/NRF2/GPX4 signalling pathway, whether other SIRT1‐related pathways are involved still needs further investigation. Finally, only aged male mice were used in this experiment and whether gender influences the experimental results also requires further verification.

## 5 Conclusion

Our study suggests that EA may improve cognitive impairment in aged mice with PND by inhibiting ferroptosis in hippocampal neurons through the SIRT1/NRF2/GPX4 pathway.

## Author Contributions

**Zhongying Du:** writing – original draft, writing – review and editing, methodology, software, formal analysis, data curation, supervision. **Binsen Zhang:** writing – review and editing, methodology, software, supervision. **Tianren Chen:** writing – review and editing, methodology, validation. **Chang Lei:** data curation. **Lu Tang:** investigation. **Sasa Yang:** formal analysis. **Chunai Wang:** funding acquisition, project administration, supervision.

## Funding

This work was supported by National Natural Science Foundation of China (Grant 82260973) and Lanzhou Municipal Science and Technology (Grant 2022‐3‐31).

## Conflicts of Interest

The authors declare no conflicts of interest.

## Data Availability

The data that support the findings of this study are available on request from the corresponding author. The data are not publicly available due to privacy or ethical restrictions.
